# Associations Between Isokinetic Knee Strength at Different Angular Velocities and Explosive Jump Performance in Young Female Athletes: A Pilot Study

**DOI:** 10.3390/jfmk11020237

**Published:** 2026-06-13

**Authors:** Daniela Falat Leütterová, Jaroslav Sučka

**Affiliations:** 1 Department of Music, Art and Physical Education, Faculty of Education, University of Presov, 080 01 Presov, Slovakia; daniela.leutterova@unipo.sk; 2 Department of Educology of Sports, Faculty of Sport, University of Presov, 080 01 Presov, Slovakia

**Keywords:** athletic training, individual sports, Humac Norm, Chronojump, peak torque, hamstring-to-quadriceps ratio, muscle asymmetry, neuromuscular performance, monitoring

## Abstract

**Background:** Isokinetic strength of the knee joint represents a significant determinant of athletic performance and injury prevention; however, its relationship with explosive performance in young female athletes remains insufficiently explored. The aim of the study was to analyze the relationships between isokinetic strength of the knee joint at different angular velocities and explosive jumping performance in young female athletes. **Methods:** The research sample consisted of 13 young female athletes enrolled in sport-oriented educational programs specializing in athletics. Explosive lower-limb power was assessed using performance tests for countermovement jump (CMJ), countermovement jump free arms (CMJ FAs) and squat jump (SJ) administered with the Chronojump system. Isokinetic strength of the knee flexors and extensors was assessed using the Humac Norm dynamometer in the concentric mode at angular velocities of 60°/s, 180°/s, and 300°/s. Peak torque, the ipsilateral H:Q ratio, and bilateral asymmetries were evaluated. Pearson’s correlation coefficient was used to analyze the relationships between the investigated parameters. **Results:** The strongest relationships with explosive performance were observed for hamstring strength at an angular velocity of 180°/s, where significant high correlations were identified with performance in the CMJ (r = 0.693), CMJ FA (r = 0.754), and SJ (r = 0.713). In contrast, quadriceps strength demonstrated predominantly low to moderate associations with jumping performance, while no significant correlations were confirmed at an angular velocity of 300°/s. Bilateral asymmetries of the knee extensors and flexors were generally low, ranging approximately between 7 and 10%, whereas the values of the ipsilateral H:Q ratio were within the physiological range of approximately 50–55%. **Conclusions:** The results suggest that the ability to generate force at higher contraction velocities, particularly in the hamstrings, is significantly associated with explosive performance in young female athletes. At the same time, isokinetic strength assessment appears to be an appropriate tool for evaluating muscular strength, muscle balance, and potential asymmetries in youth sports. However, explosive performance cannot be explained solely by the level of maximal muscular strength, but rather by a complex interaction of neuromuscular and biomechanical factors.

## 1. Introduction

Isokinetic strength of the knee joint represents a significant determinant of athletic performance and injury prevention [1]. At the same time, it is considered a reliable and objective tool for the assessment of muscular strength and function, as it enables the analysis of balance between agonist and antagonist muscle groups through strength ratios [2,3]. Compared with conventional strength assessment methods, such as one-repetition maximum testing, field-based performance tests, or isometric dynamometry, isokinetic dynamometry enables the assessment of muscle strength under controlled movement velocity throughout the entire range of motion. This allows for a more precise evaluation of peak torque, agonist–antagonist muscle balance, and inter-limb asymmetries while minimizing the influence of movement technique and momentum [2,4].

An important aspect of isokinetic strength evaluation is the use of different angular velocities, which provide distinct information regarding the neuromuscular characteristics of performance [5]. Thus, the angular velocity of testing represents a key factor, as it influences not only the values of strength parameters but also the strength of the relationship between isokinetic strength and explosive performance [6].

In explosive sports, including athletics, lower-limb muscular strength represents a key determinant of athletic performance, as it underlies the ability to generate high levels of force within a short time interval. Athletes participating in performance-oriented sports achieve higher values of peak torque, highlighting the importance of isokinetic parameters for explosive performance [7]. In Athletics, lower-limb isokinetic strength has been shown to be significantly associated with performance-related parameters [8,9].

Isokinetic strength of the knee joint muscles is significantly associated with explosive lower-limb performance, with this relationship being most observed in tasks such as the countermovement jump (CMJ) and squat jump (SJ). Although SJ is typically performed from a starting knee flexion angle of approximately 90°, CMJ allows a self-selected countermovement depth, resulting in greater variability in knee joint angles during the movement. A dominant role is played by the knee extensors, particularly the musculus quadriceps femoris, which demonstrate a stronger association with explosive performance compared with the flexors [6]. The importance of isokinetic strength for jumping performance is further supported by other studies demonstrating its association with vertical jump height [9,10]. The strongest relationships are often observed at higher angular velocities. At the same time, explosive lower-limb power represents one of the main determinants of athletic performance, as it is closely associated with the ability to generate force within a short time interval [11]. The relationship between knee joint isokinetic strength parameters and vertical jump height therefore provides important insights into the mechanisms underlying explosive performance [12]. The hamstring-to-quadriceps ratio (H:Q ratio) is commonly used as an indicator of muscle balance around the knee joint and is considered an important parameter in both performance assessment and injury prevention. In concentric isokinetic testing, values ranging approximately between 0.50 and 0.60 are generally considered physiological, whereas lower values may indicate insufficient hamstring contribution to knee stabilization and a potentially increased risk of injury [2,13].

Female athletes exhibit distinct neuromuscular characteristics, including a greater reliance on activation of the musculus quadriceps femoris, which may contribute to an increased risk of lower-limb injuries [7]. Specifically, hamstring strength in females is often lower compared with males, leading to an imbalance between the agonist and antagonist muscle groups of the knee joint. This neuromuscular pattern is associated with increased quadriceps activation and relatively lower hamstring involvement in knee stabilization, thereby increasing anterior shear forces acting on the tibia, subsequently elevating the load on the anterior cruciate ligament (ACL), and contributing to a 4–6-fold higher risk of ACL injury in women [14]. Despite the growing interest in female athletic performance, women remain underrepresented in sports science research. Most studies investigating the relationship between muscular strength, neuromuscular function, and athletic performance have been conducted in male athletes or mixed-sex populations, particularly in team sports such as soccer, basketball, and volleyball. Consequently, knowledge regarding the relationship between isokinetic strength and explosive performance in young female athletes remains limited. Furthermore, only a small number of studies have specifically examined these associations in athletics, despite the central role of explosive strength in sprinting and jumping events [15,16].

In the context of youth sports, particularly during early school age, the literature indicates a lack of studies analyzing the relationship between the level of explosive lower-limb power and the Isokinetic Strength of the knee extensors. Although research [17] has examined the relationships between various types of vertical jumps and isokinetic strength, it also emphasizes that jump execution technique plays a significant role. Movement technique is closely related to the level of neuromuscular coordination, which changes dynamically during growth and development [18,19,20]. In explosive lower-limb movements, technique substantially influences the efficiency of force production, with movement coordination and segmental body alignment playing a key role [21]. In young female athletes, this relationship is more complex due to the influence of biological maturation and neuromuscular development, suggesting that the interaction between strength, technique, and performance represents an area that remains insufficiently explored. The relationship between isokinetic strength and performance parameters such as explosive power is not consistently defined in the literature [5]. Explosive lower-limb power represents a key determinant of athletic performance, particularly in the context of acceleration and starting speed [22,23]. Nevertheless, study results show considerable variability depending on sport type, age, and measurement methodology [6]. Some studies report moderate to strong relationships between isokinetic strength and jump performance [24,25,26], whereas others indicate low or non-significant correlations [27,28], or have failed to demonstrate any relationship [29,30]. In addition, angular velocity used in isokinetic testing appears to play an important role [25,28].

Despite the widespread use of isokinetic assessment, normative data on strength profiles in female athletes remain limited, and research focusing on young female athletes and the relationship between isokinetic strength measured at different angular velocities and explosive performance remains insufficiently explored [16]. Therefore, the aim of this study is to analyze the relationships between knee joint isokinetic strength at different angular velocities (60°/s, 180°/s, 300°/s) and explosive jumping performance in young female athletes, with the aim of contributing to a better understanding of neuromuscular determinants of performance in the early stages of athletic development. It was hypothesized that higher levels of isokinetic strength, particularly at higher angular velocities, would be significantly associated with explosive jump performance.

## 2. Materials and Methods

### 2.1. Participants

The sample consisted of 13 young female athletes enrolled in extended sport-oriented educational programs primarily focused on athletics training. These groups of girls within the sports population are considered physically talented and performance-oriented individuals. The selected participants engaged in compulsory physical and sports education at least twice per week, together with additional sports training three times per week focused on Athletics.

The participants were involved in systematic athletics training primarily focused on sprint and speed-strength disciplines. The study sample consisted of athletes specializing in sprinting, hurdling, and long jump events. Athletes participating in endurance disciplines were not included in the study. Despite differences in event specialization, these disciplines can be assumed to have similar neuromuscular demands, particularly with respect to the development of explosive strength and speed-strength capabilities of the lower limbs.

Diagnostic measurements were conducted at the beginning of the competitive season, ensuring the acquisition of baseline data on the physical preparedness of the young athletes prior to the start of the season. This timeframe was intentionally selected to provide a basis for individualized optimization of the training process and to enable the identification of potential deficiencies in the preparedness of the young athletes that could influence in-season performance. Table 1 presents the basic demographic data of the study sample.

### 2.2. Procedures

The diagnostics were conducted in the morning hours. First, laboratory assessment of somatic parameters and body composition was performed. Body height was measured using a digital stadiometer BSM 170 (Biospace Co., Ltd., Seoul, Republic of Korea), while body mass and body composition parameters were assessed using direct multifrequency bioelectrical impedance analysis with the InBody 720 device (Biospace Co., Ltd., Seoul, Republic of Korea).

Prior to explosive power testing, the young athletes performed a 5 min warm-up on a cycle ergometer at a load of 50 W and a cadence of 70–80 rpm, followed by an individual warm-up consisting of dynamic stretching. The warm-up concluded with sport-specific exercises comprising three sets of three jumps performed without arm swing, with arm swing, and from a squat position. Lower-limb explosive power, with jump height as the outcome variable, was assessed using the Chronojump diagnostic system (Bosco system, Barcelona, Spain). Explosive lower-limb power was evaluated using three test types: countermovement jump without arm swing (CMJ), countermovement jump with arm swing (CMJ FA), and squat jump (SJ). To assess lateral asymmetries in lower-limb explosive power, unilateral tests were also performed, including vertical jumps executed separately on each lower limb. For each test type, the athletes performed three individual jumps, with the highest recorded performance used for analysis. A minimum rest interval of 10 s was provided between jumps.

**Figure 1:**
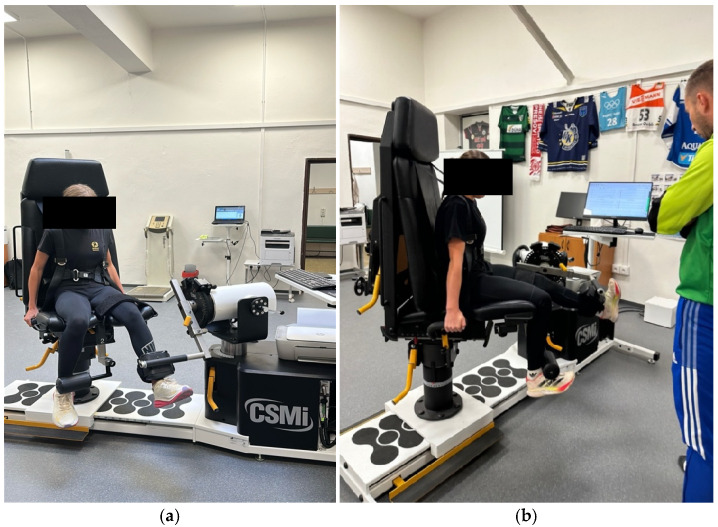
Human Norm: (**a**) flexion position; (**b**) extension position.

Assessment of knee flexor and extensor strength was conducted using the Humac Norm isokinetic dynamometer (Cybex NORM^®^, Humac, Stoughton, MA, USA), as shown in Figure 1. Isokinetic testing was performed in concentric mode at angular velocities of 60°/s, 180°/s, and 300°/s. Peak torque of the knee extensors (PTE) and flexors (PTF) was evaluated, with absolute values originally obtained in Newton-meters (Nm) subsequently normalized to relative values expressed per kilogram of body mass (Nm/kg) to account for inter-individual differences. Ipsilateral hamstring-to-quadriceps (H:Q) ratio and bilateral differences in knee extensor and flexor strength were also assessed. The ipsilateral H:Q ratio represents the percentage ratio between the anterior and posterior thigh muscle groups on the same lower limb [31]. and is calculated according to the equation:

(1) $$H\text{:}Q=\frac{hamstring\;muscle\;torque}{quadriceps\;muscle\;torque}\times 100\;(\%)$$

The bilateral difference expresses the percentage difference between corresponding segments of both lower limbs [31]. Authors [32] report a high correlation coefficient for isokinetic dynamometry methodology (ICC = 0.90–0.98), as well as a high level of reliability for the assessment of strength asymmetry ratios (3.2–8.7%) and muscle torque values (4.3–7.7%).

Testing began with individual seat adjustment based on somatic characteristics, and the lever arm of the isokinetic dynamometer was also individually configured. The axis of rotation of the lever arm was aligned with the lateral femoral epicondyle. The range of motion was set from 0° to 90°, with full knee extension at 0° defined as anatomical zero. Prior to testing, the trunk and thigh of the tested limb were stabilized using straps to eliminate undesired movements. During the test, participants held the device handles. Gravity correction for the dynamometer lever arm and the lower-limb segment was applied within the software and automatically calculated by the device.

Participants were allowed to choose voluntarily which lower limb they would start with. The dominant lower limb was defined as the preferred limb for take-off, i.e., the preferred jumping leg. Prior to testing, each participant performed five familiarization trials at low angular velocity with moderate to submaximal intensity. Subsequently, and in accordance with the recommendations of study [33] testing was conducted from the slowest to the fastest angular velocities. The testing protocol consisted of two maximal-effort repetitions in concentric mode at 60°/s and three maximal-effort repetitions at 180°/s and 300°/s. Between individual testing velocities, participants had a 2 min passive rest interval [34].

### 2.3. Statistics

Due to the relatively small sample size, this study should be interpreted as a pilot investigation providing preliminary insights into the relationship between isokinetic strength and explosive performance in young female athletes.

Given the normal distribution of data confirmed by the Shapiro–Wilk test, the arithmetic mean was used as a measure of central tendency, while variability was expressed using standard deviation (SD), minimum and maximum values, and the 95% confidence interval (95% CI). To examine the relationship between isokinetic strength and explosive performance, peak torque values of knee extensors and flexors were aggregated as the sum of both lower limbs (L + R), representing the overall strength potential of the lower extremities, and subsequently analyzed in relation to jump performance tests (CMJ, SJ, CMJ FA). Pearson’s correlation coefficient was used to determine the degree of association between variables. The strength of correlations was interpreted according to the following scale: r < 0.3—small; 0.3 ≤ r < 0.5—moderate; and r ≥ 0.5—high [35]. Statistical significance was set at *p* < 0.05. The research procedures were conducted in accordance with the ethical standards of the Declaration of Helsinki. The study was approved by the Ethics Committee for Research at the University of Prešov in Prešov (ECUP 092024PO, 2024).

## 3. Results

The results of lower-limb explosive power assessment are presented in Table 2. The highest values were recorded in the countermovement jump with free arms (CMJ FA), whereas the lowest values were observed in the squat jump (SJ). In unilateral vertical jump tests, no substantial differences were found between the dominant and non-dominant lower limbs.

The results of knee flexor and extensor isokinetic strength assessment are presented in Table 3. Higher peak torque values were recorded for the knee extensors compared with the flexors, while a progressive decrease in force production was observed with increasing angular velocity.

The H:Q ratio values (Table 4) were relatively balanced between the dominant and non-dominant lower limbs across all tested angular velocities, with no substantial deviations observed. Similarly, bilateral differences (Table 5) between the dominant and non-dominant lower limbs were generally low for both knee extensors and flexors, indicating a relatively symmetrical strength profile in the young female athletes.

The results of the correlation analysis (Table 6) demonstrated varying levels of association between the examined variables depending on the muscle group and the angular velocity used in the isokinetic testing. Stronger relationships with explosive performance were identified for the knee flexors, particularly at moderate-to-high angular velocities, whereas at very high angular velocity the correlations markedly weakened. Knee extensor strength showed predominantly low to moderate associations with vertical jump performance. Significant strong correlations were observed between hamstring peak torque at 180°/s and CMJ, CMJ FA, and SJ performance.

## 4. Discussion

### 4.1. Main Findings

The results of this study indicate a significant relationship between lower-limb isokinetic strength and the level of explosive power in young female athletes. The most important finding was a strong correlation between hamstring isokinetic strength at an angular velocity of 180°/s and performance in vertical jumps: CMJ (r = 0.693), CMJ FA (r = 0.754), and SJ (r = 0.713). These results suggest that the ability to produce force at higher contraction velocities is strongly associated with explosive performance in Athletics events.

The strong correlations observed at 180°/s further indicate that moderate-to-high contraction velocities may best reflect the neuromuscular demands of dynamic athletic performance. At the same time, the relationship between explosive power and quadriceps strength was weaker, particularly at the high angular velocity of 300°/s, where no significant correlations were observed. These findings therefore suggest that, in dynamic performance, the ability to efficiently generate force at velocities closer to those encountered in sport-specific movements may be more closely associated with explosive performance than maximal strength produced at low contraction velocities.

### 4.2. Jump Performance and Biomechanical Interpretation

This finding is also consistent with the obtained vertical jump values, which represent a simple and commonly used tool for assessing lower-limb muscular power [36,37,38]. Mean CMJ values in the studied sample exceeded 30 cm, which according to [39] corresponds to the level of moderately to highly trained adolescent female athletes. At the same time, higher values were recorded in CMJ FA compared with the standard CMJ and SJ, indicating effective utilization of take-off mechanics and coordination between upper and lower limbs. Current biomechanical research suggests that arm swing increases vertical impulse and optimizes the sequential activation of individual segments of the musculoskeletal system, thereby contributing to higher jump performance [40]. Other studies report that strength and power parameters in vertical jumping are significantly influenced by athletic specialization, with sprinters and jumpers achieving higher levels of explosive strength and performance compared with other athletic populations [41]. These findings support our results and suggest that both isokinetic strength and explosive performance are closely linked to the specific demands of individual sport disciplines.

### 4.3. Comparison with the Literature and Normative Values and Muscle Balance and Asymmetry

The relative isokinetic strength values of the quadriceps and hamstrings in the studied sample were generally consistent with normative data reported in the literature for trained youth athletes and young female football players. At the same time, the expected decline in force production at higher angular velocities (180°/s and 300°/s) was confirmed, reflecting the force–velocity relationship [30]. The results of the sample also demonstrated a relatively low level of bilateral asymmetries in both knee extensors and flexors, with mean values ranging approximately between 7 and 10%. These values are below thresholds that are frequently associated in the literature with an increased injury risk and may indicate good neuromuscular preparedness of the athletes. Reference [42] reports that athletes with greater muscular asymmetries exhibited a higher incidence of lower-limb injuries, with asymmetries between the dominant and non-dominant limbs considered a significant risk factor.

The ipsilateral H:Q ratio in the studied sample ranged approximately between 50 and 55%, which corresponds to recommended values reported for concentric testing conditions.

### 4.4. Injury Risk and Practical Implications

The literature states that a low H:Q ratio may reduce the ability of the hamstrings to stabilize the knee joint and increase loading on the anterior cruciate ligament (ACL). H:Q values below 0.60 at 60°/s may indicate an increased risk of muscle injuries and ACL-related injuries [43]. Although the H:Q ratio has traditionally been considered an important injury risk indicator, recent systematic reviews highlight that the H:Q ratio alone is not sufficient for injury prediction. As an isolated parameter, it has limited predictive value, and injury risk should be interpreted in the context of multiple factors such as inter-limb asymmetries, training load, fatigue, and neuromuscular control [13]. Therefore, the H:Q ratio by itself is not a sufficient predictor of injury; however, when combined with the assessment of bilateral asymmetries and neuromuscular control, it represents an important indicator of muscular balance and potential injury risk. Interesting findings are also reported by [44], who showed that athletes with a moderate (5–7 years) or short (<5 years) training age exhibited higher levels of knee extensor and flexor strength asymmetry compared with athletes with more than 8 years of training experience. Younger athletes tend to demonstrate greater asymmetry due to insufficient compensatory adaptations, which may be associated with the absence of neuromuscular strategies developed through specialized training. Muscle strength and balance are key factors in the prevention of lower-limb injuries, as confirmed by studies [45]. Significant muscular imbalance may increase discomfort and lead to a higher risk of injury, as reported by the study conducted by [46]. In young football players, the assessment of asymmetries and their subsequent correction before the onset of the growth spurt is considered an effective strategy to reduce asymmetries and thereby lower injury risk in later stages of development [47].

Given the observed association between hamstring strength at 180°/s and jump performance, coaches may consider incorporating training methods aimed at improving high-velocity force production, such as sprinting, sprint drills, plyometric exercises, hurdle jumps, bounding exercises, and ballistic resistance training. These methods may better reflect the neuromuscular demands of sprinting and jumping disciplines than traditional slow-velocity strength training alone.

### 4.5. Multifactorial Nature of Performance

Another important aspect is the fact that athletic performance cannot be explained solely by the level of maximal muscle strength. Several studies indicate that performance in sprinting, agility, and repeated sprint tasks is more strongly influenced by dynamic and plyometric abilities than by maximal strength assessed through isokinetic testing. Study [48] reported only low to moderate correlations between lower-limb isokinetic strength and sprint performance, suggesting that maximal strength alone is not the dominant determinant of athletic performance. Therefore, explosive performance appears to be associated with a complex interaction of rate of force development, neuromuscular coordination, and biomechanical movement efficiency.

### 4.6. Limitations

When interpreting the results, it is also necessary to consider the limited number of studies conducted in female youth populations. Study [15] reports that only a small proportion of sports science research is carried out on women, while adaptations to strength training may be to some extent sex-specific. For this reason, results obtained in young female athletes should be interpreted with caution and often compared with analogous athletic populations.

Nevertheless, the findings of this study suggest that isokinetic strength may represent a valuable tool for assessing muscular strength, muscle balance, and explosive performance in young female athletes, with the most important determinant of explosive performance appearing to be the ability to produce force at higher contraction velocities that are closer to the dynamics of real sporting movement.

The most important limitation of the study was the relatively small sample size (*n* = 13), which may reduce the statistical robustness of the observed associations and limit the generalizability of the findings. In addition, the sample consisted exclusively of young female athletes participating in sprint- and power-oriented athletics disciplines, which further limits the applicability of the findings to other athletic populations. Another limitation was the use of concentric-only isokinetic testing and the absence of sprint performance assessments, which would have allowed a more comprehensive evaluation of the relationship between muscle strength and athletic performance. Therefore, the present findings should be interpreted as preliminary and pilot in nature, and their confirmation requires further research conducted on larger and more homogeneous samples of young female athletes.

A further limitation of the study was the absence of biological maturation assessment. Variables such as age at menarche, Tanner stage, or predicted adult height were not evaluated, although maturation status may substantially influence strength and explosive performance in adolescent female athletes. Future studies should consider incorporating maturation-related variables to improve the interpretation of neuromuscular performance outcomes.

Given the exploratory nature of the study and the relatively small sample size, multiple correlation analyses were performed without formal correction for multiple comparisons. Therefore, the possibility of Type I error cannot be excluded and the findings should be interpreted as preliminary. Future studies with larger samples should confirm these associations using more robust statistical approaches.

Another limitation of the study is that the research protocol was not prospectively registered. Although prospective registration is not routinely required for observational cross-sectional studies, its implementation may improve transparency and reproducibility of research procedures and statistical analyses. Future studies should consider prospective registration of study protocols.

The exclusive inclusion of female athletes limits the generalizability of the findings and precludes comparisons between sexes. In addition, the cross-sectional design provides information only at a single time point and does not allow the stability of the observed relationships to be evaluated. Future longitudinal studies should examine the development of these relationships over time.

## 5. Conclusions

In conclusion, the results of this study identified significant association between lower-limb isokinetic strength and explosive performance in young female athletes, with hamstring force production at higher contraction velocities, particularly at 180°/s, showing the strongest associations with jumping performance. At the same time, relatively low levels of bilateral asymmetries and physiological H:Q ratio values were observed, which may indicate good muscular balance and neuromuscular preparedness in the studied sample.

The findings also support the importance of isokinetic assessment as an accurate tool for evaluating muscular strength, asymmetries, and potential injury risk factors in youth sports. Nevertheless, it is necessary to emphasize that explosive athletic performance is a multifactorial phenomenon that is not determined solely by maximal muscle strength, but also by the ability for rapid force production, neuromuscular coordination, movement biomechanics, and efficient use of elastic energy during the take-off phase.

From a practical perspective, the results suggest that developing the speed-strength capabilities of the hamstrings may be beneficial for explosive performance in young female athletes. They also underscore the value of regular assessment of asymmetries and muscle balance in youth athletic training.

Given the relatively small sample size, these findings should be interpreted with caution and considered preliminary. Further research involving larger samples and longitudinal study designs is required before the present findings can be generalized to broader populations of young athletes.

## Figures and Tables

**Table 1:** Demographic characteristics of the study sample.

	Age	Training Age	BH (cm)	BW (kg)	PBF (%)	SMM (%)
Mean ± SD	13.1 ± 0.7	4.2 ± 1.6	162.0 ± 5.6	46.9 ± 7.6	13.7 ± 5.9	47.0 ± 3.5
Min–Max	11.8–14.3	2.1 ± 7.8	150.9–169	33.9–67.0	3.1–24.7	41.9–53.6

Note: Training age—duration of systematic training; BH—body height; BW—body weight; PBF—percentage of body fat; and SMM—percentage of skeletal muscle mass.

**Table 2:** Level of explosive strength of the lower limbs.

	CMJ	CMJ FA	SJ	DL	NL
Mean ± SD	30.4 ± 4.8	33.7 ± 5.5	26.9 ± 3.8	15.3 ± 2.6	15.4 ± 2.5
Min–Max	22.1–42.1	27.2–48.8	21.7–36.4	11.3–20.5	11.0–20.2
95% CI	27.5–33.2	30.3–36.9	24.6–29.2	13.7–16.9	13.9–16.9

Note: CMJ—countermovement jump; CMJ FA—countermovement jump with free arm movement; SJ—squat jump; DL—dominant leg; NL—non-dominant leg; SD—standard deviation; Min—minimum; Max—maximum; and 95% CI—95% confidence interval.

**Table 3:** Level of strength of knee flexors and extensors.

	Dominant Leg			Non-Dominant Leg		
	PTE 60°/s	PTE 180°/s	PTE 300°/s	PTE 60°/s	PTE 180°/s	PTE 300°/s
Mean ± SD	2.44 ± 0.31	1.73 ± 0.19	1.48 ± 0.29	2.40 ± 0.33	1.68 ± 0.21	1.44 ± 0.30
Min–Max	1.65–2.83	1.27–2.09	1.24–2.41	1.57–2.78	1.22–2.02	0.77–2.16
95% CI	2.25–2.63	1.61–1.85	1.30–1.66	2.20–2.60	1.55–1.81	1.26–1.63
	PTF 60°/s	PTF 180°/s	PTF 300°/s	PTF 60°/s	PTF 180°/s	PTF 300°/s
Mean ± SD	1.28 ± 0.20	0.94 ± 0.17	0.75 ± 0.19	1.23 ± 0.22	0.91 ± 0.17	0.73 ± 0.17
Min–Max	0.75–1.53	0.61–1.28	0.36–1.09	0.76–1.58	0.59–1.22	0.36–1.04
95% CI	1.16–1.40	0.84–1.04	0.64–0.87	1.09–1.36	0.80–1.01	0.63–0.84

Note: PTE—peak torque of the quadriceps; PTF—peak torque of the hamstrings; 60°/s, 180°/s, 300°/s—angular velocities of isokinetic testing; SD—standard deviation; Min—minimum; Max—maximum; and 95% CI—95% confidence interval.

**Table 4:** Ipsilateral ratio of knee flexors and extensors.

	Dominant Leg			Non-Dominant Leg		
	H:Q 60°/s	H:Q 180°/s	H:Q 300°/s	H:Q 60°/s	H:Q 180°/s	H:Q 300°/s
Mean ± SD	52.4 ± 4.8	54.5 ± 7.3	51.6 ± 11.8	51.5 ± 8.1	54.2 ± 10.1	51.4 ± 8.9
Min–Max	45.0–62.0	45.0–70.0	29.0–73.0	36.0–65.0	34.0–74.0	35.0–65.0
95% CI	49.5–55.3	50.1–58.9	44.5–58.8	46.6–56.3	48.1–60.3	45.9–56.8

Note: H:Q—conventional ipsilateral hamstring-to-quadriceps ratio (%); 60°/s, 180°/s, 300°/s—angular velocities of isokinetic testing; SD—standard deviation; Min—minimum; Max—maximum; and 95% CI—95% confidence interval.

**Table 5:** Bilateral ratio of knee flexors and extensors.

	Q:Q 60°/s	Q:Q 180°/s	Q:Q 300°/s	H:H 60°/s	H:H 180°/s	H:H 300°/s
Mean ± SD	7.3 ± 5.9	6.9 ± 4.8	7.9 ± 10.6	9.1 ± 8.6	8.4 ± 12.2	9.9 ± 6.9
Min–Max	1.0–20.0	0.0–17.0	0.0–40.0	0.0–28.0	0.0–45.0	2.0–23.0
95% CI	3.8–10.9	4.0–9.8	1.5–14.4	3.8–14.3	1.1–15.7	5.7–14.1

Note: Q:Q—bilateral ratio of knee extensors (%); H:H—bilateral ratio of knee flexors (%); 60°/s, 180°/s, 300°/s—angular velocities of isokinetic testing; SD—standard deviation; Min—minimum; Max—maximum; and 95% CI—95% confidence interval.

**Table 6:** Correlation matrix of the observed variables.

	CMJ	CMJ FA	SJ
PTE 60°/s	0.439 (95% CI: −0.16–0.80)	0.428 (95% CI: −0.17–0.79)	0.541 (95% CI: −0.03–0.84)
PTE 180°/s	0.331 (95% CI: −0.28–0.74)	0.380 (95% CI: −0.22–0.77)	0.461 (95% CI: −0.13–0.81)
PTE 300°/s	0.030 (95% CI: −0.53–0.58)	−0.025 (95% CI: −0.57–0.53)	−0.094 (95% CI: −0.61–0.48)
PTF 60°/s	0.432 (95% CI: −0.17–0.79)	0.488 (95% CI: −0.10–0.82)	0.462 (95% CI: −0.13–0.81)
PTF 180°/s	0.693 * (95% CI: 0.22–0.90)	0.754 * (95% CI: 0.35–0.92)	0.713 * (95% CI: 0.26–0.91)
PTF 300°/s	0.361 (95% CI: −0.24–0.76)	0.365 (95% CI: −0.24–0.76)	0.202 (95% CI: −0.40–0.68)

Note: CMJ—countermovement jump; CMJ FA—countermovement jump with free arm movement; SJ—squat jump; PTE—peak torque of the quadriceps; PTF—peak torque of the hamstrings; 60°/s, 180°/s, 300°/s—angular velocities of isokinetic testing; 95% CI—95% confidence interval; and * *p* < 0.05.

## Data Availability

The original data presented in this study are openly available in Zenodo at https://zenodo.org/records/20160681 (accessed on 10 June 2026).

## Abbreviations

The following abbreviations are used in this manuscript:

CMJ	Countermovement jump
CMJ FAs	Countermovement jump free arms
SJ	Squat jump
DL	Dominant lower limb
NL	Non-dominant lower limb
H:Q	Hamstring-to-quadriceps ratio (ipsilateral ratio)
Q:Q	Bilateral quadriceps ratio
H:H	Bilateral hamstring ratio
BH	Body height
BW	Body weight
PBF	Percentage of body fat
SMM	Skeletal muscle mass
SD	Standard deviation
PTE	Peak torque of knee extensors
PTF	Peak torque of knee flexors
CI	Confidence interval

## References

[1] Lv J.-F., Ao Y.-X., Luo W.-L., Xiao F.-J., Liufu Y.-F., Yang J.-X. (2026). Three Decades of Research on Knee Isokinetic Strength: A Bibliometric and Visual Exploration of Global Trends and Emerging Frontiers. J. Orthop..

[2] Cozette M., Leprêtre P.-M., Doyle C., Weissland T. (2019). Isokinetic Strength Ratios: Conventional Methods, Current Limits and Perspectives. Front. Physiol..

[3] Duarte J.P., Valente-dos-Santos J., Coelho-e-Silva M.J., Couto P., Costa D., Martinho D., Seabra A., Cyrino E.S., Conde J., Rosado J. (2018). Reproducibility of isokinetic strength assessment of knee muscle actions in adult athletes: Torques and antagonist-agonist ratios derived at the same angle position. PLoS ONE.

[4] Dvir Z. (2025). Isokinetics: Muscle Testing, Interpretation and Clinical Applications.

[5] Sung J.-Y., Lee K.-L., Noh K.-W., Park S. (2026). Isokinetic Knee Strength Profiles, Conventional Hamstring-to-Quadriceps Ratio, and Performance Decrement in Weightlifting and Wrestling Athletes: A Cross-Sectional Study. Sci. Rep..

[6] Kabacinski J., Szozda P.M., Mackala K., Murawa M., Rzepnicka A., Szewczyk P., Dworak L.B. (2022). Relationship between Isokinetic Knee Strength and Speed, Agility, and Explosive Power in Elite Soccer Players. Int. J. Environ. Res. Public Health.

[7] Freire R., Huff D., Butterick B., Figueroa E.C., Siegler J.C. (2025). Knee Isokinetic Strength Benchmarks in Athletes across Sports Categories and Performance Levels. Biol. Sport.

[8] Şahin İ.H., Kıvrak A.O. Analysis of the Phases of Sprint in Terms of Isokinetic Leg Strength, Anaerobic Endurance, and Balance. https://www.jomh.org/articles/10.22514/jomh.2025.127?utm_source=chatgpt.com.

[9] Kural B., Çağlar E.Ç., Akkuş Uçar M., Özer U., Yentürk B., Çayır H., Çelik N.M., Çimen E., Arıkan G., Ceylan L. (2025). Isokinetic Knee Strength as a Predictor of Performance in Elite Ski Mountaineering Sprint Athletes. Medicina.

[10] Sattler T., Sekulic D., Esco M.R., Mahmutovic I., Hadzic V. (2015). Analysis of the Association between Isokinetic Knee Strength with Offensive and Defensive Jumping Capacity in High-Level Female Volleyball Athletes. J. Sci. Med. Sport.

[11] Chen L., Zhang Z., Huang Z., Yang Q., Gao C., Ji H., Sun J., Li D. (2023). Meta-Analysis of the Effects of Plyometric Training on Lower Limb Explosive Strength in Adolescent Athletes. Int. J. Environ. Res. Public Health.

[12] Westwood C., Welbeck A., Killelea C., Howard P., Faherty M., Le D., Zerega R., Reiter C.R., Sell T.C. (2025). Examining Isokinetic Knee Peak Torque and Time to Peak Torque as Predictors of Vertical Jump Height in Division I Men’s Basketball Players. PLoS ONE.

[13] Kellis E., Sahinis C., Baltzopoulos V. (2023). Is Hamstrings-to-Quadriceps Torque Ratio Useful for Predicting Anterior Cruciate Ligament and Hamstring Injuries? A Systematic and Critical Review. J. Sport Health Sci..

[14] Hewett T.E., Ford K.R., Hoogenboom B.J., Myer G.D. (2010). Understanding and Preventing Acl Injuries: Current Biomechanical and Epidemiologic Considerations—Update 2010. N. Am. J. Sports Phys. Ther..

[15] Stanković M.N., Čaprić I.M., Katanić B.D., Radaković R.Ž., Murić B.B., Kahrović I.H., Jelaska I.D. (2025). Effects of different strength training on sprint, jump and strength performance in female soccer players. A systematic review. Med. Dello Sport.

[16] Risberg M.A., Steffen K., Nilstad A., Myklebust G., Kristianslund E., Moltubakk M.M., Krosshaug T. (2018). Normative Quadriceps and Hamstring Muscle Strength Values for Female, Healthy, Elite Handball and Football Players. J. Strength Cond. Res..

[17] Harčarik G., Falat Leütterová D. (2024). The Relationship between Isokinetic Knee Flexion and Squat Jump Performance. J. Kinesiol. Exerc. Sci..

[18] Watkins C.M., Barillas S.R., Wong M.A., Archer D.C., Dobbs I.J., Lockie R.G., Coburn J.W., Tran T.T., Brown L.E. (2017). Determination of Vertical Jump as a Measure of Neuromuscular Readiness and Fatigue. J. Strength Cond. Res..

[19] Cleather D., Cushion E. (2019). Muscular Coordination during Vertical Jumping. J. Hum. Perform. Health.

[20] Andraos Z. (2025). Comprehensive Assessment and Individualized Training Guidance in Vertical Jump Performance: From Force–Velocity Profiling to Neuromuscular Diagnostics. MOJSM.

[21] Vončina T., Pori P., Šarabon N., Spudić D. (2024). Vpliv tehnične izvedbe na višino vertikalnega enono-žnega skoka z nasprotnim gibanjem pri rokometaših. Rev. Šport.

[22] Haugen T.A., Tønnessen E., Seiler S. (2012). Speed and Countermovement-Jump Characteristics of Elite Female Soccer Players, 1995-2010. Int. J. Sports Physiol. Perform..

[23] Hammami M., Negra Y., Shephard R.J., Chelly M.S. (2017). The Effect of Standard Strength vs. Contrast Strength Training on the Development of Sprint, Agility, Repeated Change of Direction, and Jump in Junior Male Soccer Players. J. Strength Cond. Res..

[24] Tsiokanos A., Kellis E., Jamurtas A., Kellis S. (2002). The Relationship between Jumping Performance and Isokinetic Strength of Hip and Knee Extensors and Ankle Plantar Flexors. Isokinet. Exerc. Sci..

[25] Saliba L., Hrysomallis C. (2001). Isokinetic Strength Related to Jumping but Not Kicking Performance of Australian Footballers. J. Sci. Med. Sport.

[26] Pua Y.-H., Koh M.T.-H., Teo Y.-Y. (2006). Effects of Allometric Scaling and Isokinetic Testing Methods on the Relationship between Countermovement Jump and Quadriceps Torque and Power. J. Sports Sci..

[27] Alemdaroğlu U. (2012). The Relationship Between Muscle Strength, Anaerobic Performance, Agility, Sprint Ability and Vertical Jump Performance in Professional Basketball Players. J. Hum. Kinet..

[28] Iossifidou A., Baltzopoulos V., Giakas G. (2005). Isokinetic Knee Extension and Vertical Jumping: Are They Related?. J. Sports Sci..

[29] González-Ravé J.M., Juárez D., Rubio-Arias J.A., Clemente-Suarez V.J., Martinez-Valencia M.A., Abian-Vicen J. (2014). Isokinetic Leg Strength and Power in Elite Handball Players. J. Hum. Kinet..

[30] Cometti G., Maffiuletti N.A., Pousson M., Chatard J.C., Maffulli N. (2001). Isokinetic Strength and Anaerobic Power of Elite, Subelite and Amateur French Soccer Players. Int. J. Sports Med..

[31] Dauty M., Potiron-Josse M., Rochcongar P. (2003). Identification of Previous Hamstring Muscle Injury by Isokinetic Concentric and Eccentric Torque Measurement in Elite Soccer Player. Isokinet. Exerc. Sci..

[32] Impellizzeri F.M., Bizzini M., Rampinini E., Cereda F., Maffiuletti N.A. (2008). Reliability of Isokinetic Strength Imbalance Ratios Measured Using the Cybex NORM Dynamometer. Clin. Physiol. Funct. Imaging.

[33] Wilhite M.R., Cohen E.R., Wilhite S.C. (1992). Reliability of Concentric and Eccentric Measurements of Quadriceps Performance Using the KIN-COM Dynamometer: The Effect of Testing Order for Three Different Speeds. J. Orthop. Sports Phys. Ther..

[34] Rahnama N., Lees A., Bambaecichi E. (2005). Comparison of Muscle Strength and Flexibility between the Preferred and Non-Preferred Leg in English Soccer Players. Ergonomics.

[35] Cohen J. (1988). Statistical Power Analysis for the Behavioral Sciences.

[36] Helgerud J., Engen L.C., Wisloff U., Hoff J. (2001). Aerobic Endurance Training Improves Soccer Performance. Med. Sci. Sports Exerc..

[37] Kotzamanidis C., Chatzopoulos D., Michailidis C., Papaiakovou G., Patikas D. (2005). The Effect of a Combined High-Intensity Strength and Speed Training Program on the Running and Jumping Ability of Soccer Players. J. Strength Cond. Res..

[38] Siegler J., Gaskill S., Ruby B. (2003). Changes Evaluated in Soccer-Specific Power Endurance Either with or without a 10-Week, in-Season, Intermittent, High-Intensity Training Protocol. J. Strength Cond. Res..

[39] Markovic G. (2007). Does Plyometric Training Improve Vertical Jump Height? A Meta-Analytical Review. Br. J. Sports Med..

[40] Cefai C.M., Shaw J.W., Cushion E.J., Cleather D.J. (2024). An Arm Swing Enhances the Proximal-to-Distal Delay in Joint Extension during a Countermovement Jump. Sci. Rep..

[41] Philpott L.K., Forrester S.E., van Lopik K.A., Hayward S., Conway P.P., West A.A. (2021). Countermovement Jump Performance in Elite Male and Female Sprinters and High Jumpers. Proc. Inst. Mech. Eng. Part P J. Sports Eng. Technol..

[42] Lehance C., Binet J., Bury T., Croisier J.L. (2009). Muscular Strength, Functional Performances and Injury Risk in Professional and Junior Elite Soccer Players. Scand. J. Med. Sci. Sports.

[43] Bhatt J., D’Onofrio R., Padasala M., Joksimović M., Bruno C., Melino D., Manzi V. (2018). Muscle Injuries in Athletes. The Relationship between H/Q Ratio (Hamstring/Quadriceps Ratio). Ital. J. Sports Rehabil. Posturol..

[44] Fousekis K., Tsepis E., Vagenas G. (2010). Lower Limb Strength in Professional Soccer Players: Profile, Asymmetry, and Training Age. J. Sports Sci. Med..

[45] Fernández-Baeza D., Diaz-Urena G., González-Millán C. (2022). Effect of an Individualised Training Programme on Hamstrings and Change Direction Based on Tensiomyography in Football Players. Appl. Sci..

[46] Junge N., Morin J.-B., Nybo L. (2023). Leg Extension Force-Velocity Imbalance Has Negative Impact on Sprint Performance in Ball-Game Players. Sports Biomech..

[47] Read P.J., Oliver J.L., Myer G.D., De Ste Croix M.B.A., Lloyd R.S. (2018). The Effects of Maturation on Measures of Asymmetry During Neuromuscular Control Tests in Elite Male Youth Soccer Players. Pediatr. Exerc. Sci..

[48] İnce İ., Tortu E. (2025). Association between Intra and Inter-Limb Strength Asymmetry with Sprint Kinematics and Force-Velocity Profile in Youth Team Athletes. Knee.

